# Acute Alcohol Intoxication With Accompanying Neurovascular Decline in Young Adults: A Sobering Case Series

**DOI:** 10.7759/cureus.11572

**Published:** 2020-11-19

**Authors:** Christ Ordookhanian, Ryan F Amidon, Paul Kaloostian

**Affiliations:** 1 Emergency Medicine, University of California Riverside School of Medicine, Riverside, USA; 2 Neuroscience, University of California Riverside, Riverside, USA; 3 Neurological Surgery, Riverside Community Hospital, Riverside, USA; 4 Neurological Surgery, Paul Kaloostian MD Inc, Riverside, USA

**Keywords:** cerebrovascular, cerebrovascular injury, stroke, alcohol misuse, ethanol intoxication, case series, ethanol induced, emergent conditions

## Abstract

With an increase in both popularity and incidence, young adults continue to consume increasing amounts of ethanol-containing beverages at alarmingly short intervals, at times, far surpassing the threshold of binge drinking. From mixed beverages to party delights, high-concentration ethanol consumption continues to wreak havoc on the health of the younger generation. In this dual case series, we discuss two unique cases of alcohol-induced neurovascular compromise following episodes of high-volume ethanol consumption and acute neurovascular decline with varying outcomes. These cases highlight the hyperacute onset of severe pathology and the dire need for immediate medical intervention. While outcomes vary from case to case, our findings are congruent with those of vast medical literature that supports the consensus that immediate intervention to restore neurovascular flow is crucial for desired outcomes. At the end of the day, we cannot control the amount of alcohol that enters the mouths of our patients, but rather, we can educate them on safer practices while highlighting the risk and life-changing consequences of such risky behavior.

## Introduction

The likelihood of enduring a cerebrovascular complication increases exponentially with age. It is commonly seen in older adults but its occurrence is not ruled out in young adults, children, and even infants, often resulting in significant morbidity and mortality [1]. While unfortunate to encounter, the pediatric incidence of arterial ischemic infarctions is quite low, with the highest reported incidence value being 0.008% [2]. In adults younger than 45 years of age, the incidence rises to 0.0113% of the population [2]. While these numbers are low, there has been an upward trend in ischemic strokes since the early 1980s, which can be attributed to not only rises in cases but also the ability to detect subacute infarctions via advanced neuroimaging and risk stratification by an individual’s known risk factors [3].

While ischemic cerebrovascular events are the most common etiology of stroke within its general class, when presenting in young adults, the etiology is often that of a congenital origin, vascular pathology, hematological condition, underlying electrolyte imbalance, or ingestion of toxins such as alcohol or illicit drugs [4-5]. There has been an understanding within the general public, which holds true in a large number of published studies, that light alcohol consumption can decrease the risk of an ischemic stroke. Recent evidence contraindicating these findings is noted; however, this notion is well disbursed within the general public and often brought up by patients [6]. In the setting of heavy alcohol consumption, the risk for all major types of strokes is increased, particularly those of ischemic etiology [7]. Alcohol has been reported to induce vasoconstriction and precipitate the formation of aneurysms and microlesions within the cerebral arteries of experimental animals [7]. Of worthy mention, alcohol intoxication leading to impaired balance is also associated with an increase in head trauma that alone can precipitate coagulation, embolization, and autonomic disturbances and promote the likelihood of a cerebrovascular event from occurring [7]. In patient population studies, patients within the medium and higher-risk cohorts of ischemic strokes were found to be intoxicated 45%-50% of the time [8]. This highlights the risk that alcohol consumption plays as a precipitating factor for cerebrovascular events.

Within these two patient case series, we highlight dual cases in which patients with a high-risk factor associated with heavy alcohol consumption presented with an advanced neurological compromise and significant neuroimaging defects, leading to the need for emergent neurosurgical intervention. While ischemic brain infarcts are unlikely in our case patient population, we report two patient stories where the advanced stage of the neurological deficit, the gross pathological changes, and the overall prognosis is of the utmost severity amongst those in literature today.

## Case presentation

Patient #1

**Figure 1:**
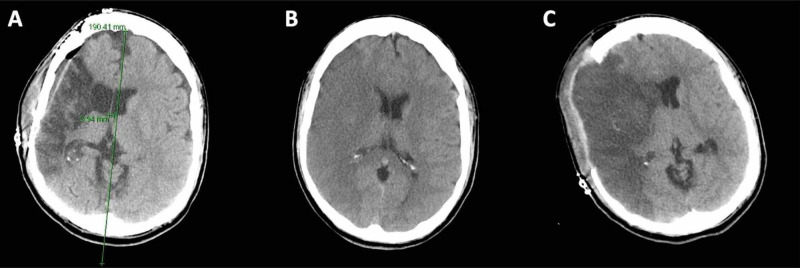
(A) CT reveals a 3.94 mm midline shift postoperatively with a clear right MCA infarct. (B) CT reveals a region of hypodensity indicative of malignant right-sided MCA infarct. (C) Post-decompressive surgery CT scan showing a large malignant MCA infarct with less mass effect. CT: Computed tomography MCA: Middle cerebral artery

A 30-year-old male presented to the emergency department (ED) approximately 11 hours after beginning a night’s worth of heavy binge alcoholic consumption, per the historical context provided by the transporting roommate. During the exam, the patient appeared obtunded, mildly arousable, with a gaze preference, and left-sided hemiplegia. A head computed tomography (CT) scan and CT angiography (CTA) scan both revealed right-Right MCA thrombosis with a large right-sided middle cerebral artery (MCA) region infarction with a minor anterior cerebral artery (ACA) regional infarct, along with a 3 mm midline shift (Figures 1a-1c).

The patient's past medical, family, and surgical history were all non-contributory. Laboratory complete blood count (CBC), complete metabolic panel (CMP), and coagulation factors were all within normal limits. There was a pertinent negative finding of no prior drug use as reported by the patient. Serum alcohol levels were 300 mg/dl at the time of presentation to the ED. Initial medical management of 3% sodium chloride (NaCl) did not improve the patient’s clinical course. At this time, surgical intervention with emergent right hemicraniectomy was indicated to decompress the brain and relieve intracranial pressure. The postoperative course was notable for the resolution of acute presenting symptoms and an improvement from 1/5 strength on the left side to 3/5, allowing for assisted ambulation.

Patient #2

**Figure 2:**
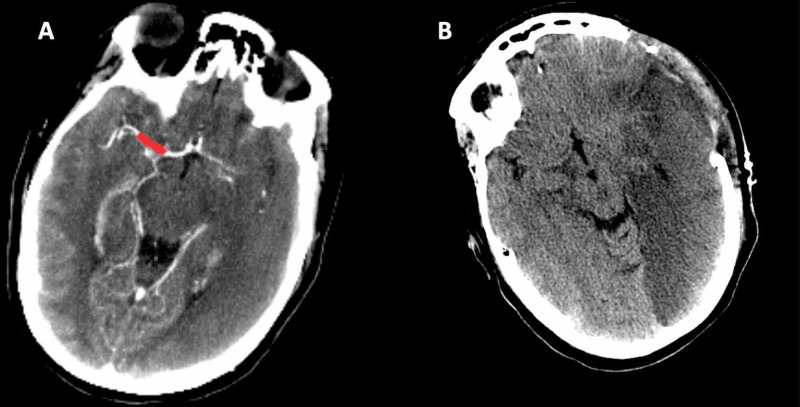
(A) CTA reveals acute thrombosis of left M1 segment with large left MCA malignant infarct. (B) Post-hemicraniectomy CT showing malignant left MCA infarct with craniectomy noted. CTA: Computed tomography angiography CT: Computed tomography MCA: Middle cerebral artery

A 31-year-old female patient transferred to our care from an outside hospital with global aphasia and right-sided hemiplegia, with onset after reported heavy binge alcohol consumption along with concurrent heavy tobacco use. There was no evidence indicating other drug use per the endorsement of the patient. Upon arrival, the patient underwent CT and CT angiography (CTA) scans of the head, which revealed left middle cerebral artery (MCA) infarction with left M1 thrombosis with notable cerebral edema and herniation (Figures 2a-2b).

Per the consult of the on-call neurology service, tissue plasminogen activator (tPA) and 3% sodium chloride (NaCl) were administered in hopes of resolving symptomology, which failed and, in fact, caused the rapid deterioration of the patient’s neurological status. Emergent surgical hemicraniectomy was indicated and performed, which revealed severe cerebral edema. The postoperative course was unchanged with no clinically significant improvement of the patient’s aphasia and right-sided hemiplegia.

## Discussion

Strokes are divided into two broad categories: ischemic and hemorrhagic. In a hemorrhagic stroke, blood volume is overabundant within the cranium, whereas an ischemic stroke is characterized by reduced blood supply and oxygen to a region of the brain [9]. A plethora of factors may increase the risk for the onset of stroke, from electrolyte imbalance to alcohol consumption [4-5]. Alcohol, or ethanol, is a known risk factor for many diseases and complications. Our cases demonstrate ischemic strokes precipitated by high-dose ethanol consumption or binge-drinking (BD). BD is conventionally defined as consuming five or more alcoholic drinks for men, or four or more drinks for women, in a single drinking session. While it is well-established that BD increases the risk of cerebral infarction, many studies find that low-dose ethanol consumption may reduce the risk of ischemic stroke while still increasing the risk of hemorrhagic complication [10].

Unfortunately, studies examining the relationship between ethanol ingestion and ischemic stroke are frequently contradictory. For instance, Sacco et al. argued that high-dose ethanol consumption was a valid determinant of ischemic stroke recurrence [11]. In contrast, Gorelick et al. argued that acute ethanol ingestion may not represent an independent risk factor for ischemic stroke, whereas hypertension and smoking were independent risk factors [12]. Their study involved middle-aged and geriatric patients. Furthermore, they explained that the common association of alcohol consumption with smoking represented a confound when attempting to correlate acute ethanol ingestion with cerebral infarct. On the other hand, Hillbom et al. determined that smoking was not an independent risk factor in young people, as the negative effects of smoking on arterial vessel walls were dependent on the amount and duration of smoking [13]. This would explain why Gorelick et al. found smoking to be a stronger determinant of brain infarction in middle-aged and geriatric patients. Examining a younger patient population would, therefore, reduce the risk of results being impacted by the aforementioned potential confound. Other drug use associated with BD may constitute an additional confound.

The ethanol-associated risk of ischemic stroke and accompanying prognosis are commonly found to be dose-dependent. Using a mouse model, Xu et al. demonstrated worse outcomes in the high-dose group (2.8 g/kg/day; peak blood ethanol concentration of 37 mM) than the low/moderate-dose group (0.7 g/kg/day; peak blood ethanol concentration of 9 mM) after suffering brain ischemia/reperfusion injuries [10]. Relative to the low/moderate-dose group, the high-dose group experienced greater post-ischemic neutrophil infiltration, microglial activation, blood-brain barrier (BBB) breakdown, and stimulation of pro-inflammatory cytokines/chemokines. On the other hand, the authors observed neuroprotective effects in the low/moderate-dose group.

The body’s post-ischemic inflammatory response is complex with many variables, including inflammation, oxidative stress, and apoptosis. Following the onset of ischemia, adhesion molecules on endothelial cells are upregulated to enable neutrophil infiltration into the ischemic brain [10]. Simultaneously, microglia activate inflammatory processes, recruiting matrix metallopeptidases (MMPs), cytokines, and chemokines. Activated MMPs facilitate neutrophil invasion and BBB breakdown. Pro-inflammatory cytokines/chemokines expand the scope of this response by stimulating apoptotic events, activating microglia, upregulating adhesion molecules, and facilitating neutrophil infiltration. Dead cells release reactive oxygen species (ROS) and adenosine triphosphate (ATP), further activating microglia [10]. This pro-inflammatory response, which is exacerbated by high-dose ethanol, is associated with worsened outcomes post-ischemic stroke.

Glutamate excitotoxicity is a second method by which ethanol affects post-ischemic prognosis. Zhao et al. believed that N-methyl-D-aspartate (NMDA) excitotoxicity may explain the disparate effects of low-dose and high-dose ethanol [14]. They found that an upregulation of NMDA receptors, precipitated by chronic high-dose ethanol ingestion, facilitated post-ischemic glutamate excitotoxicity. This was accompanied by a downregulation of excitatory amino acid transporter 2 (EAAT2), which normally regulates extracellular glutamate concentration via reuptake, resulting in an overabundance of synaptic glutamate. Contrarily, low-dose ethanol was found to upregulate EAAT2. Acute high-dose ethanol also upregulated EAAT2 and inhibited NMDA receptors. The authors described the discrepancy between acute and chronic high-dose ethanol effects as a neuroadaptive response. Nicotinamide adenine dinucleotide phosphate (NAD(P)H) oxidase-mediated oxidative stress is a third mechanism mediated by ethanol exposure. Zhao et al. demonstrated in a rat model that chronic ethanol ingestion upregulated NAD(P)H oxidase in the cerebral cortex and arteries post-ischemic stroke [15]. Under ischemic conditions, NAD(P)H oxidase produces ROS, which induces neuronal death, increasing the infarct volume. Altura and Altura responded to studies that demonstrate neuroprotective benefits in low-dose ethanol ingestion using animal models by noting that the majority of animal studies use anesthetics and other drugs, which affect cerebral vascular smooth muscle, interact with ethanol, and impact cerebral blood flow [16]. Therefore, the apparent differences between low/moderate-dose and high-dose conditions may be influenced by a confound.

Mechanisms by which ethanol may induce ischemic stroke include endothelial dysfunction and the acceleration of atherosclerosis. When the endothelium is compromised, it fails to appropriately regulate vascular function, including vasoconstriction and vasodilation. Goslawski et al. demonstrated how ethanol may promote endothelial dysfunction by increasing vasoconstriction and altering nitric oxide (NO) bioavailability [17]. They found that young binge drinkers acquired vasodilation deficits mirroring those in patients who drank six drinks per day for more than eight years. In the BD group, endothelin-1-induced vasoconstriction was significantly heightened relative to the alcohol-abstaining group. Endothelin-1 may reduce NO production and promote its degradation, further impairing vasodilation, as NO mediates vasodilation. The authors explained that the link between BD and endothelial dysfunction may explain why BD is associated with accelerated atherosclerosis, which constitutes the most common cause of ischemic stroke [17].

Altura and Altura found evidence of another mechanism by which ethanol may induce hypoxia and ischemic stroke: cerebral vascular spasms [16]. Their experimental design was most promising, avoiding many of the confounds that may impact the results of similar studies. Arterioles and venules were directly examined after removing portions of the cranium in a bloodless and atraumatic vascular field, avoiding vascular injury, using a TV-image intensification microscope recording system. Ventilation and respiration were controlled and arterial blood gases and pH were monitored. Blood flow from the arteriole to venule was assessed using a local carbon clearance technique. The application of ethanol (of 10-500 mg/dl concentrations), using any form of administration, induced graded contractile responses in the cerebral microvasculature. Blood ethanol concentrations over 300 mg/dl induced irreversible arteriolar spasms and sometimes focal hemorrhages. Vasoconstriction of venules was also observed in a dose-dependent manner: 0.01-1% ethanol reduced vessel diameter by 8%-60%, doses above 0.3% induced irreversible spasm and rupture. These changes reduced blood flow from arteriole to venule, creating hypoxia. Furthermore, the authors found that blood ethanol concentration as little as 37 mg/dl induced contraction; this concentration appears in humans after consuming under one ounce of whiskey. Intense spasm of the cerebral vasculature, and potentially focal hemorrhaging, occurred with blood ethanol concentrations over 250 mg/dl. The authors concluded that prolonged cerebral vascular spasm could result in cerebral ischemia and hemorrhage [16]. The restriction of cerebral blood flow may also induce hypertension, which is considered a risk factor for stroke [12]. Upon review of the aforementioned studies, it is clear that BD both induces ischemic stroke and worsens post-infarct prognosis.

Treatment of ethanol-induced ischemic stroke is a complex and time-sensitive process. The conventional approach to acute stroke management includes intravenous thrombolysis with recombinant tissue plasminogen activator (rtPA) within 4.5 hours of symptom onset unless contraindicated [18]. The function of tissue plasminogen activator (tPA) is to dissolve blood clots to improve blood flow, which induces reperfusion. While reperfusion may worsen brain injury, earlier reperfusion, rather than later, typically improves prognosis [10,18]. Hyperosmolar therapy, via hypertonic saline solution, is commonly used in post-ischemic stroke management, especially in patients suffering from cerebral edema. The most commonly used hypertonic saline solution is 3% NaCl administered as a continuous infusion. Other accepted standards of care for cerebral edema include elevating the head of the bed, short-term ventilation, and sedation [19]. When medical management is ineffective, surgical decompression to relieve intracranial pressure may be indicated.

Cerebral edema represents a complex pathology that must be resolved. Under ischemic conditions, aerobic metabolism transitions to anaerobic metabolism, which limits the amount of energy that cells have to regulate intracellular ion concentrations [19]. As intracellular sodium abnormally rises, water enters the cells, swelling them; simultaneously, abnormally high intracellular calcium levels trigger apoptosis. These events characterize cytotoxic edema, which can lead to a growing mass effect, inducing cerebral ischemia and, potentially, herniation. Hyperosmolar therapy seeks to reduce cellular swelling via dehydration, but as our modern understanding of cerebral edema and treatment remains lacking, this does not always work.

The cases described in this series represent severe cases of ethanol-induced ischemic stroke; however, unidentified concomitant drug use may confound this etiology. The blood ethanol level of Patient 1 was 300 mg/dl, which is beyond the 250 mg/dl threshold described by Altura and Altura for inducing intense spasm of cerebral vasculature [16]. Both patients experienced MCA infarction, which is associated with the inefficacy of medical management [19]. Thrombolysis-induced reperfusion via tPA in Patient 2 may have led to the deterioration in neurological status. After medical management failed to improve both patients’ clinical courses, surgery was indicated. Ethanol-induced ischemic stroke can be life-threatening. Fortunately, both patients survived after the emergent intervention; however, lasting complications remained.

## Conclusions

As ethanol consumption continues to be a plague within all our communities today, we must pay particular attention to our younger populations, as binge drinking is often practiced fearlessly. Our dual case series aims to address two rare and severe outcomes of ethanol consumption where excess intake resulted in a severe cerebrovascular compromise, requiring surgical intervention, and a long post-operative course to restore full ambulatory function. In both cases, patients presented with an acute cerebrovascular compromise with cerebral edema and a worsening neurological state. Rapid medical intervention supplemented by surgical management was able to alleviate gross pathology with the hope of eventually restoring full ambulation. Both patients were unfortunately lost to follow-up.
